# Clinical and prognostic implications of high-sensitivity cardiac troponin T concentrations in type 2 non-ST elevation myocardial infarction

**DOI:** 10.1016/j.ijcha.2022.100972

**Published:** 2022-02-12

**Authors:** K.M. Eggers, T. Baron, A. Gard, B. Lindahl

**Affiliations:** a Department of Medical Sciences, Uppsala University, Uppsala, Sweden; b Department of Medical Sciences and Uppsala Clinical Research Center, Uppsala University, Uppsala, Sweden

**Keywords:** CAD, Coronary artery disease, CVD, Cardiovascular disease, cTn, Cardiac troponin, hs-cTnT, High-sensitivity cardiac troponin T, ICD-10-CM, International Classification of Diseases, 10th revision, Clinical Modification, MAE, Major adverse events, MI, Myocardial infarction, NSTEMI, Non-ST elevation myocardial infarction, SWEDEHEART, Swedish Web-system for Enhancement and Development of Evidence-based care in Heart disease Evaluated According to Recommended Therapies, TOTAL-AMI, Tailoring Of Treatment in All comers with Acute Myocardial Infarction, High-sensitivity cardiac troponin T, Type 2 myocardial infarction, Risk prediction

## Abstract

**Background:**

While the clinical importance of cardiac troponin is well-known in type 1 myocardial infarction (MI), evidence on this topic in type 2 MI is limited. We assessed the clinical and prognostic implications of high-sensitivity cardiac troponin (hs-cTnT) concentrations in a large sample of patients with type 2 MI.

**Methods:**

Retrospective registry-based cohort study (SWEDEHEART) including 4607 patients with type 2 MI and 43,405 patients with type 1 MI, used for comparisons. Patients with ST-elevation MI were excluded. Multivariable-adjusted regressions were applied to investigate the associations of hs-cTnT concentrations (highest measured value during each hospitalization) with clinical variables and prognosis during a median follow-up of up to 1.9 years.

**Results:**

Hs-cTnT concentrations (median 264 [25th, 75th percentiles 112–654] ng/L) were significantly associated with various cardiovascular risk factors and comorbidities in type 2 non-ST elevation MI (NSTEMI) but only weakly with the underlying triggering condition. Most of these findings including the magnitude of hs-cTn release were similar to type 1 NSTEMI. Hs-cTnT (ln) independently predicted all-cause mortality (hazard ratio 1.13 [95% confidence interval 1.09–1.17]) and major adverse events (hazard ratio 1.13 [95% confidence interval 1.10–1.17]) in type 2 NSTEMI, similar as for type 1 NSTEMI according to interaction analysis. The associations of hs-cTnT (ln) with poor prognosis tended to be stronger in type 2 NSTEMI patients without known cardiovascular disease.

**Conclusions:**

Hs-cTnT concentrations independently predict adverse outcome in type 2 NSTEMI. The similarities to type 1 NSTEMI however, are striking and emphasize the difficulty to distinguish both MI types.

## 1 Introduction

Type 2 myocardial infarction (MI) is defined as acute myocardial injury associated with ischemic symptoms or ECG changes that is triggered by a condition other than coronary plaque disruption or coronary intervention and causes oxygen supply/demand mismatch [1]. Compared to patients with type 1 MI, those with type 2 MI tend to be older and suffer more frequently from comorbidities, both cardiovascular and non-cardiovascular [2]. Cardiac troponin (cTn) concentrations however, tend to be lower in type 2 MI [3], [4], [5] indicating less severe myocardial injury. Still, type 2 MI patients have similar or even higher risk of adverse outcome as those with type 1 MI when taking into account differences in risk panorama [2], [6]. This indicates that, compared to type 1 MI, prognosis in type 2 MI to a stronger degree may be determined by residual risk. Even variations in the association of cTn with risk between both MI types may contribute. However, information on the clinical and prognostic implications of cTn concentrations in type 2 MI and relative to type 1 MI is limited [5].

The aims of the present analysis were thus, 1) to assess the prognostic importance of high-sensitivity cardiac troponin T (hs-cTnT) concentrations in type 2 MI, 2) to investigate predictors of hs-cTnT concentrations in type 2 MI, and 3) to compare these results with findings from patients with type 1 MI.

## 2 Material and methods

### 2.1 Study population

This study is part of the TOTAL-AMI (Tailoring Of Treatment in All comers with Acute Myocardial Infarction) project. The primary aim of TOTAL-AMI is to study the mechanisms and implications of different MI subtypes [1] and of comorbidities (e.g. chronic obstructive pulmonary disease, atrial fibrillation, renal dysfunction) in MI. TOTAL-AMI uses data from SWEDEHEART (Swedish Web-system for Enhancement and Development of Evidence-based care in Heart disease Evaluated According to Recommended Therapies). SWEDEHEART is a nationwide registry prospectively collecting data from patients admitted to Swedish coronary care units or other specialized facilities because of suspected acute coronary syndrome. This registry provides information on patient demographics, medical history, symptoms, physical and ECG findings upon admission, blood test results including the highest cTn value measured during the hospitalization, coronary status, in-hospital management, and discharge diagnoses. Upon hospital admission, patients receive information about SWEDEHEART, have the right to deny participation and get their data erased upon request. Written informed consent is not required according to Swedish law.

The cohort of interest for the present study included all patients discharged with a primary diagnosis of type 2 MI between January 2010 and May 2018. Only the first registered MI during the study period was considered. We included only patients assessed with hs-cTnT measurements and excluded those in whom other cTn assays or other biomarkers of myocardial injury had been used. We also excluded patients with ST-elevation MI since serial cTn measurements are not always performed in this condition. Accordingly, only patients with non-ST elevation MI (NSTEMI) had been studied. A cohort of patients with a primary diagnosis of type 1 NSTEMI selected using the same criteria was used for comparative purposes.

All data had been made pseudonymized before the statistical analyses. The study was conducted according to the principles of the 1975 Declaration of Helsinki and had been approved by the Regional Ethical Review Board in Stockholm (2012/60-31/2).

### 2.2 Diagnostic classification and definitions

The diagnoses recorded in SWEDEHEART are set by the treating physicians at each respective hospital based on the Universal Definition and its subclassifications [1], as recommended within the SWEDEHEART framework [7].

Triggering conditions contributing to type 2 NSTEMI were retrospectively defined using the following criteria, adopted from [8]:

– Tachyarrhythmia: non-sinus rhythm and heart rate > 150/min;
– Bradycardia: heart rate < 30/min;
– Hypertension: systolic blood pressure > 180 mmHg or systolic blood pressure > 160 mmHg and pulmonary edema;
– Hypotension: systolic blood pressure < 90 mmHg or cardiogenic shock in case of absence of anemia;
– Anemia: hemoglobin ≤ 90 g/L in males, ≤80 g/L in females;
– Respiratory causes: admission because of dyspnea together with a primary or secondary diagnosis of respiratory disease (International Classification of Diseases, 10th revision, Clinical Modification [ICD-10-CM] codes J00-99) and absence of pulmonary rales upon admission.

In case of multiple trigger conditions being present in a single patient, a decision on the most appropriate condition was made depending on the primary or secondary discharge ICD-10-CM code.

Prevalent coronary artery disease (CAD) was defined as a history of MI or coronary revascularization, angiographic evidence of coronary stenosis > 50% or coronary revascularization performed during the index hospitalization. Prevalent cardiovascular disease (CVD) was defined as CAD, a history of stroke or peripheral artery disease.

### 2.3 Prognostic evaluation

Information on patient outcome was obtained by merging SWEDEHEART with data from the Swedish Population Registry (data on the vital status of all Swedish residents) and the Swedish Patient Registry (hospitalization dates and discharge diagnoses based on ICD-10-CM codes), the latter held by the Swedish Board of Health and Welfare. The outcomes considered for this analysis were all-cause mortality and major adverse events (MAE), defined as the composite of all-cause mortality, hospitalization for recurrent MI (ICD-10-CM code I21), heart failure (ICD-10-CM code I50) or ischemic stroke (ICD-10-CM code I63). Outcomes were counted from admission to the coronary care unit until an event occurred or until end of follow-up. For all-cause mortality, this was May 2018, and December 2017 for MAE since there is a time lag for processing hospitalization data within the Swedish Patient Registry. During the first 30 days after the index hospitalization, it is not possible to separate a new MI from the index MI in the Patient Registry. Therefore, only MI occurring at least 30 days after the hospitalization were counted to avoid ‘contamination’ from the index event.

### 2.4 Statistical analysis

All continuous variables are reported as medians with 25th and 75th percentiles with comparisons made using the Mann-Whitney *U* test. Categorical variables are expressed as frequencies and percentages.

Multiple linear regressions were used to investigate the associations of hs-cTnT concentrations in type 2 NSTEMI with clinical variables: age, sex, current smoking, hypertension, diabetes mellitus, hyperlipidemia, estimated glomerular filtration rate (CKD-EPI equation), previous MI, previous coronary revascularization, previous heart failure, previous stroke, peripheral artery disease, chronic obstructive pulmonary disease, previous or present cancer and atrial fibrillation on the admission ECG. In addition, adjustment was made for admission year and hospital. Due to positive skew, hs-cTnT values were converted to their natural logarithm (ln) before being entered into the analysis.

The associations of hs-cTnT (ln) with adverse outcome were studied using Cox regression models. We applied a similar adjustment set as for the multiple linear regressions but included also ST-segment changes on the admission ECG. Interaction analyses were applied to assess the prognostic implications of hs-cTnT (ln) relative to the type of NSTEMI (type 2 vs. type 1) and in type 2 NSTEMI subcohorts with and without CAD or CVD, respectively. Cumulative incidence curves were constructed using the Kaplan-Meier method, and the log-rank test was used to compare the occurrence of adverse outcomes across hs-cTnT quartiles.

In all tests, a two-sided p-value < 0.05 was considered significant. The software package SPSS 27.0 (SPSS Inc., Chicago, IL) was used for the analyses.

## 3 Results

Totally 121,952 unique MI patients had been admitted between January 2010 and May 2018. Of these, 81,242 patients had available results for hs-cTnT. In the remaining patients, CK-MB had been measured (n = 548), or other cTn assays (n = 40,162) had been used. After exclusion of 31,968 patients with ST-elevation MI, 450 patients with type 3–5 MI and 1401 patients with missing information on MI type, 48,012 patients with NSTEMI remained in the final dataset. Of these, 4607 (9.6%) patients had type 2 NSTEMI and 43,405 (90.4%) had type 1 NSTEMI. Data on clinical characteristics is presented in Table 1. Patients with type 2 NSTEMI tended to be older compared to those with type 1 NSTEMI, were more often female and had higher prevalence of cardiovascular risk factors and comorbidities. Totally, 1756 (38.1%) type 2 NSTEMI patients had prevalent CAD and 2267 (49.2%) had prevalent CVD. Triggering conditions could be identified retrospectively in 1379 (29.9%) type 2 NSTEMI patients based on registry data: tachyarrhythmia (n = 215 [15.9%]), bradycardia (n = 5 [0.4%]), hypertension (n = 420 [30.5%]), hypotension (n = 133 [9.6%]), anemia (n = 302 [21.9%]), respiratory causes (n = 300 [21.8%]).

**Table 1:** Clinical characteristics and crude event rates in patients with type 2 and type 1 NSTEMI.

	Type 2 NSTEMI (n = 4607)	Missing data	Type 1 NSTEMI (n = 43,405)	Missing data
*Demographics*				
Age (years)	78 (70–85)	–	72 (63–80)	–
Men	2254 (51.1%)	–	8211 (62.2%)	–

*Risk factors*				
Current smoking	608 (13.2%)	–	7667 (17.7%)	7
Hypertension	2773 (60.2%)	–	24,277 (56.0%)	20
Diabetes	1179 (25.6%)	1	10,148 (23.4%)	30
Hyperlipidemia	16,767 (36.4%)	3	13,667 (31.5%)	42
Body mass index (kg/m^2^)	25.7 (22.9–29.0)	588	26.6 (24.2–29.8)	2633
eGFR (mL/min/1.73 m^2^)	62.0 (42.6–81.1)	227	75.8 (57.5–89.3)	1132

*Comorbidities*				
Previous MI	984 (21.4%)	1	7912 (18.2%)	34
Previous PCI/CABG	741 (16.1%)	1	7333 (16.9%)	35
Heart failure	596 (12.9%)	1	2947 (6.8%)	37
Atrial fibrillation at admission	1199 (26.1%)	12	4118 (9.5%)	202
Previous stroke	572 (12.4%)	1	3544 (8.2%)	38
Peripheral artery disease	465 (10.1%)	–	2693 (6.2%)	–
COPD	814 (17.7%)	–	3286 (7.6%)	–
Previous/present cancer	315 (6.8%)	–	1569 (3.6%)	–

**Hs-cTnT concentration (ng/L)**	264 (112–654)	–	262 (98–747)	–

*Diagnostic procedures*				
Echocardiography	3164 (68.7%)	–	35,361 (81.5%)	–
Coronary angiography	1745 (37.9%)	–	35,837 (82.6%)	–

*Treatments*				
In-hospital PCI/CABG	389 (8.4%)	–	26,683 (61.5%)	–
Medications at discharge *				
Aspirin	2830 (64.8%)	11	39,183 (92.8%)	56
P2Y12 blockers	1473 (33.7%)	11	34,480 (81.7%)	56
Oral anticoagulants	785 (18.0%)	11	3713 (8.8%)	56
Betablockers	3382 (77.5%)	11	36,548 (86.6%)	56
RAAS-inhibitors	2679 (61.4%)	11	32,330 (76.6%)	56
Statins	2710 (62.1%)	11	38,066 (90.2%)	56

*Outcomes*				
All-cause mortality	2107 (45.7%)	–	9712 (22.4%)	–
Myocardial infarction	411 (9.3%)	–	3561 (8.5%)	–
Heart failure	554 (12.5%)	–	3214 (7.7%)	–
Stroke	181 (4.1%)	–	1236 (3.0%)	–
MAE	2377 (53.8%)	–	12,859 (30.9%)	–

eGFR: estimated glomerular filtration rate; MI: myocardial infarction; PCI: percutaneous coronary intervention; CABG: coronary artery bypass grafting; COPD: chronic obstructive pulmonary disease; hs-cTnT: high-sensitivity cardiac troponin T; RAAS: renin-angiotensin-aldosterone-system; MAE: major adverse events.

* Data from hospital survivors (type 2 NSTEMI: n = 4377; type 1 NSTEMI: n = 42,278).

**Fig. 1:**
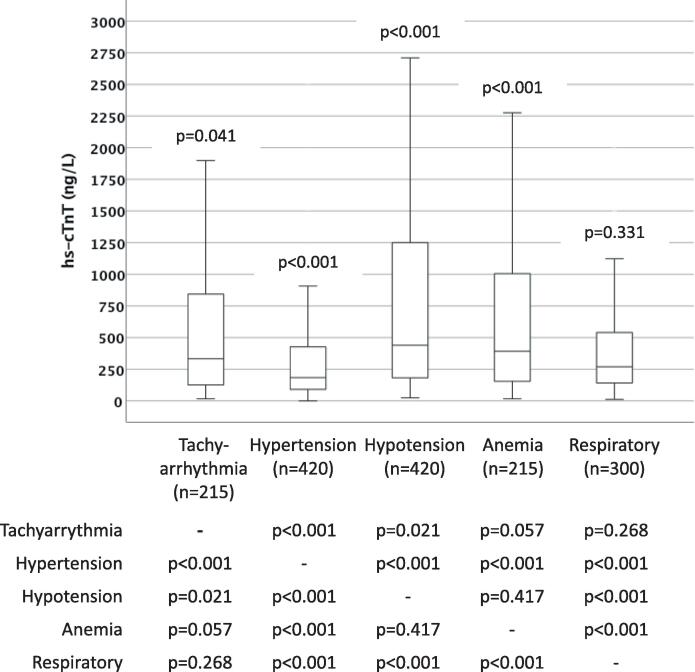
**Hs-cTnT concentrations in relation to triggering conditions in type 2 NSTEMI.** Patients with bradycardia were not considered due to their small number (n = 5). P-values on top of the respective boxplots refer to comparisons (Mann-Whitney tests) with patients without an identifiable triggering condition (n = 3228; median hs-cTnT 260 [25, 75th percentiles 108–644] ng/L). P-values for intergroup comparisons are presented below the figure.

The median hs-cTnT concentration in type 2 NSTEMI patients was 264 (25th, 75th percentiles 112–654) ng/L without significant difference to type 1 NSTEMI patients (262 [98–747] ng/L; p = 0.415). The median hs-cTnT concentrations in type 2 NSTEMI patients with and without CAD were 300 (119–773) ng/L and 244 (107–587) ng/L (p < 0.001), and 308 (123–794) ng/L and 228 (102–543) ng/L in type 2 NSTEMI patients with and without CVD (p < 0.001). Hs-cTnT concentrations in relation to triggering conditions are depicted in Fig. 1. Compared to type 2 NSTEMI patients without an identifiable triggering condition (n = 3228), hs-cTnT concentrations were higher in patients with tachyarrhythmia, hypotension and anemia, and lower in patients with hypertension.

Upon multivariable adjustment, significant associations were noted between higher hs-cTnT (ln) and increasing age, male sex, diabetes, lower estimated glomerular filtration rate, heart failure, previous stroke and peripheral artery disease (Supplemental Table). Higher hs-cTnT (ln) was also associated with the triggering condition at borderline significance when forcing this as additional variable into the model (β = 0.061; p = 0.043). Additional adjustment for ST-segment changes as explanatory variable did not affect these associations (data not shown). The pattern of variables associated with hs-cTnT (ln) was largely similar as for type 1 NSTEMI, as indicated by the overlapping 95% confidence intervals of the regression coefficients (Supplemental Table). However, hs-cTnT (ln) exhibited a weaker association with atrial fibrillation in type 2 NSTEMI compared to type 1 NSTEMI.

**Fig. 2:**
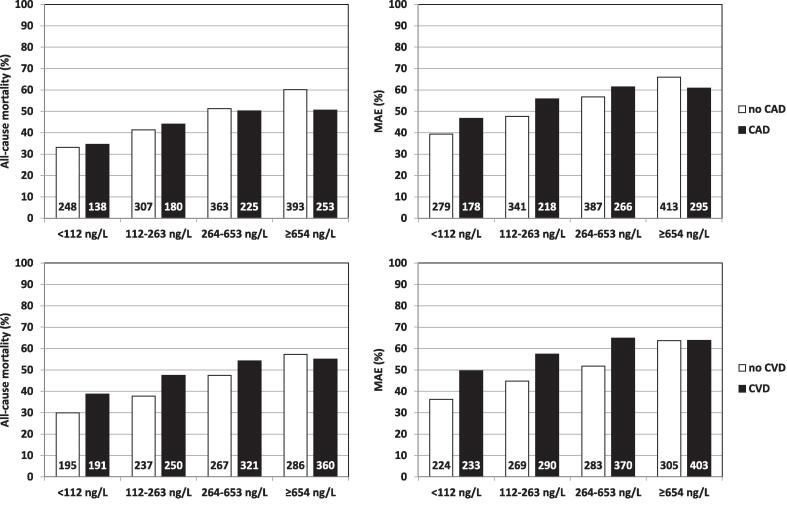
**Adverse outcome in relation to hs-cTnT quartiles in type 2 NSTEMI patients with and without coronary artery disease (upper panel) and with and without cardiovascular disease (lower panel).** The numbers at the bottom of the bars indicate numbers of patients suffering an adverse event within each cohort. MAE: major adverse event; CAD: coronary artery disease; CVD: cardiovascular disease.

Information on crude event rates is presented in Table 1. During a median follow-up of 1.9 (0.6–3.7) years, 2107 (45.7%) type 2 NSTEMI patients died. Totally 2377 (53.8%) type 2 NSTEMI patients suffered a MAE during a median follow-up of 1.5 (0.4–3.3) years. Increasing hs-cTnT quartiles were significantly associated with both outcomes with diverging Kaplan-Meier curves already early after admission (Supplemental Fig. 1, Fig. 2).

The multivariable-adjusted hazard ratios per 1-standard deviation-increase in hs-cTnT (ln) were 1.13 (95% confidence interval 1.09–1.17) regarding all-cause mortality and 1.13 (95% confidence interval 1.10–1.17) regarding MAE. Higher hs-cTnT (ln) independently predicted all components of MAE apart from stroke (Table 2). Interaction analysis indicated similar strengths of the associations with all outcomes as in type 1 NSTEMI, apart for all-cause mortality for which hs-cTnT was a slightly weaker predictor in type 2 NSTEMI. The point estimates of the hazard ratios for hs-cTnT (ln) were higher in type 2 NSTEMI patients without CAD or CVD compared to those with these conditions (Table 2). This was supported by the interactions of CVD on the association of hs-cTnT (ln) with outcome (all-cause mortality: p_interaction_ = 0.045; MAE: p_interaction_ = 0.040), apparently driven by stronger risk gradients across hs-cTnT concentrations in patients without CVD (Fig. 2). The interactions of CAD on the associations of hs-cTnT (ln) with adverse outcome were weaker (all-cause mortality: p _interaction_ = 0.270; MAE: p _interaction_ = 0.273). The triggering condition exhibited no significant interaction on the association of hs-cTnT (ln) with adverse outcome (all-cause mortality: p _interaction_ = 0.469; MAE: p _interaction_ = 0.126).

**Table 2:** Associations of hs-cTnT (ln) with adverse outcome in patients with type 2 and type 1 NSTEMI.

	Type 2 NSTEMI			Type 1 NSTEMI			
All patients	n	HR (95% CI)	p	n	HR (95% CI)	p	p int.
All-cause mortality	4492	1.13 (1.09–1.17)	<0.001	42,881	1.19 (1.17–1.21)	<0.001	0.044
MI	2496	1.12 (1.04–1.21)	0.004	32,687	1.06 (1.04–1.09)	<0.001	0.105
Heart failure	2665	1.14 (1.07–1.22)	<0.001	33,496	1.20 (1.17–1.23)	<0.001	0.883
Stroke	2389	1.02 (0.92–1.13)	0.751	32,229	1.11 (1.07–1.16)	<0.001	0.587
MAE	4316	1.13 (1.10–1.17)	<0.001	41,193	1.15 (1.13–1.16)	<0.001	0.439

*With CAD*							
All-cause mortality	1709	1.11 (1.05–1.17)	<0.001	–	–	–	–
MAE	1647	1.12 (1.06–1.18)	<0.001	–	–	–	–

*Without CAD*							
All-cause mortality	2783	1.14 (1.09–1.19)	<0.001	–	–	–	–
MAE	2669	1.14 (1.09–1.19)	<0.001	–	–	–	–

*With CVD*							
All-cause mortality	2199	1.11 (1.06–1.17)	<0.001	–	–	–	–
MAE	2115	1.12 (1.07–1.17)	<0.001	–	–	–	–

*Without CVD*							
All-cause mortality	2293	1.18 (1.12–1.24)	<0.001	–	–	–	–
MAE	2201	1.17 (1.11–1.23)	<0.001	–	–	–	–

Model adjusted for admission year, hospital, age, sex, current smoking, hypertension, diabetes, hyperlipidemia, previous myocardial infarction, previous coronary revascularization, congestive heart failure, previous stroke, ST-changes upon admission, atrial fibrillation upon admission, estimated glomerular filtration rate, chronic obstructive pulmonary disease, previous or present cancer and peripheral vascular disease, as appropriate.

HR: hazard ratio; CI: confidence interval; MI: myocardial infarction; MAE: major adverse event; CAD: coronary artery disease; CVD: cardiovascular disease.

## 4 Discussion

In this registry-based study investigating a large cohort of type 2 NSTEMI patients, several interesting observations were made. First, the magnitude of hs-cTnT release appeared to be similar as in type 1 NSTEMI. This contrasts to data reported elsewhere [3], [4], [5] and likely depends on the exclusion of patients with ST-elevation MI from our cohort. Even differences in blood sampling strategies may have contributed. Second, hs-cTnT concentrations were associated with several risk factors and comorbidities. The pattern of these entities showed many similarities with type 1 NSTEMI. The underlying triggering condition in contrast, had no apparent impact on the magnitude of hs-cTnT release in type 2 NSTEMI. Third, hs-cTnT independently predicted adverse outcome, both with respect to all-cause mortality and non-fatal events apart from stroke. Again, the overall associations were similar as for type 1 NSTEMI which is in line with recently reported data from the APACE study [5].

Our results emphasize the importance of hs-cTnT as a strong and independent predictor of prevalent comorbidities and poor outcome in type 2 NSTEMI. Accordingly, the magnitude of hs-cTnT release should not be trivialized in this condition. Our data however, also highlight the difficulties to distinguish type 2 from type 1 NSTEMI, and hs-cTnT concentrations provide no clue in this regard. Even previous data demonstrated that cTn only provides limited discriminative value [4], [5], [8], [9]. Some studies have suggested that this could be enhanced by the consideration of clinical findings and/or other biomarkers [10], [11], [12], [13]. However, none of these approaches is generally acknowledged, and the differentiation of both NSTEMI types will likely remain cumbersome, in particular in patients with critical illness or known CAD.

Given the limitations in differential diagnostic applicability, the question arises in which way hs-cTnT could help to aid management in type 2 NSTEMI. Considering the strong association with adverse outcome, high hs-cTnT concentrations emphasize the need of thorough assessment of cardiac morphologic and functional status including the presence of CAD in each individual patient. We suggest such measures to be initiated early during the hospitalization in order to provide timely care. Patients with low hs-cTnT concentrations should be managed based on their individual likelihood of having underlying cardiac disease. Diagnostic procedures could be performed in the outpatient setting, provided the absence of serious triggering conditions that require immediate management. As indicated by the results of our subgroup analysis, this applies in particular to patients without known CVD. However, we acknowledge that the clinical effects of hs-cTnT-guided management in type 2 MI are unknown as of yet.

The prognostic implications of hs-cTnT in patients with and without CVD deserve some separate comments. The risk gradients across increasing hs-cTnT concentrations were stronger in the latter cohort which contributed to greater hazard ratios with significant interaction terms. This discrepancy was driven by lower event rates in patients without CVD and low hs-cTnT concentrations but partly also by higher event rates in those without CVD but with high hs-cTnT concentrations. Type 2 NSTEMI patients without CVD presenting with low hs-cTnT concentrations appear thus, to represent a true lower-risk cohort. The high event rates in those with high hs-cTnT concentrations could be explained by a stronger contribution of non-cardiovascular conditions to outcome. An alternative and more intriguing explanation is the possible presence of undiagnosed and thus, untreated CVD in these patients. This reinforces the need of thorough in-hospital assessment in case of a pronounced hs-cTnT release.

Our study has several strengths. We assessed a large cohort of type 2 NSTEMI patients with information on several clinically relevant outcomes. Because of the unique Swedish personal identification number and mandatory health registries, we have complete information on outcome in all patients. There are also some limitations that need to be considered. This was a retrospective registry-based investigation with inherent selection bias. Patients with type 2 MI are for example, often given ward in non-cardiology facilities [14] and extrapolating our findings to these patients should be done with caution. Although all hospitals participating in SWEDEHEART are annually monitored, the data cannot be of the same quality as in a prospective study. However, the accuracy of the data and the registry has been found to be high [15]. Only the highest hs-cTnT concentration documented during each hospitalization is registered in SWEDEHEART. Accordingly, we lack information on dynamic hs-cTnT changes and true peak hs-cTnT concentrations since the data provided in SWEDEHEART may have been influenced by variations in blood sampling strategies between the hospitals. However, this will rather lead to an underestimation than an overestimation of the prognostic importance of hs-cTnT. Patients with ST-elevation MI could not be considered in our analysis since cTn is not serially measured in this setting. While the SWEDEHEART frameworks recommends the use of the Universal Definition for diagnostic classification [1], [7], some hospitals applied hs-cTnT cut-offs greater than the 99th percentile (i.e. > 30 ng/L or > 40 ng/L) during the early years of the study period and after transitioning from the conventional cTnT assay. This was the case in 302 (6.6%) type 2 NSTEMI patients and 1416 (3.3%) type 1 NSTEMI patients and may have amplified the associations of hs-cTnT concentrations with clinical data and outcome overall, but not between both MI types. We cannot exclude erroneous diagnosis or misclassification of NSTEMI in some cases since the diagnoses were set locally by the treating physicians and without formal adjudication [16]. Finally, the presence and type of triggering conditions was retrospectively classified on the basis of information available in SWEDEHEART. This resulted in missing data in a considerable proportion of patients.

Our results in conclusion, demonstrate that higher hs-cTnT concentrations are a strong and independent risk predictor in type 2 NSTEMI. The similarities to type 1 NSTEMI however, are striking, even with respect to clinical entities contributing to higher hs-cTnT concentrations. This emphasizes the difficulties to distinguish both NSTEMI types. The dimension of this problem is amplified by the fact that a substantial proportion of patients regarded as having a type 2 MI is admitted to non-cardiology wards [14]. These patients tend to be less intensively managed in terms of diagnostic procedures and cardioprotective pharmacotherapies. The findings presented here thus, highlight the need of careful clinical work-up of type 2 NSTEMI patients, in particular for those with high hs-cTnT concentrations.

## Declaration of Competing Interest

The authors declare that they have no known competing financial interests or personal relationships that could have appeared to influence the work reported in this paper.
