# Supplementary material for: Clinical and prognostic implications of high-sensitivity cardiac troponin T concentrations in type 2 non-ST elevation myocardial infarction
Source: Int J Cardiol Heart Vasc. 2022 Feb 12;39:100972. doi: 10.1016/j.ijcha.2022.100972 (PMC8843950; doi:10.1016/j.ijcha.2022.100972)
Supplement: Supplementary data 1 [file mmc1.docx]

**SUPPLEMENTAL MATERIAL**

**Supplemental Table. Predictors of hs-cTnT (ln) in patients with type 2 and type 1 NSTEMI.**

**Supplemental Figure 1. Cumulative incidence of all-cause mortality in relation to hs-cTnT quartiles in patients with type 2 NSTEMI.**

**Supplemental Figure 2. Cumulative incidence of major adverse events in relation to hs-cTnT quartiles in patients with type 2 NSTEMI.**

**Supplemental Table. Predictors of hs-cTnT (ln) in patients with type 2 and type 1 NSTEMI.**

|  | **Type 2 NSTEMI (n=4366)** | | | **Type 1 NSTEMI (n=42,092)** | | |
| --- | --- | --- | --- | --- | --- | --- |
|  |  | | |  | | |
|  |  | | |  | | |
|  | **β** | **B (95% CI)** | **p value** | **β** | **B (95% CI)** | **p value** |
|  |  |  |  |  |  |  |
|  |  |  |  |  |  |  |
| Age (10 years) | 0.085 | 0.095 (0.058 to 0.133) | <0.001 | 0.048 | 0.056 (0.042 to 0.070) | <0.001 |
| Male sex | 0.073 | 0.187 (0.112 to 0.262) | <0.001 | 0.069 | 0.206 (0.177 to 0.234) | <0.001 |
| Current smoking | 0.020 | 0.073 (-0.040 to 0.187) | 0.203 | 0.052 | 0.190 (0.154 to 0.227) | <0.001 |
| Hypertension | -0.016 | -0.042 (-0.120 to 0.036) | 0.295 | -0.037 | -0.105 (-0.134 to 0.077) | <0.001 |
| Diabetes | 0.040 | 0.115 (0.027 to 0.204) | 0.010 | 0.019 | 0.064 (0.031 to 0.097) | <0.001 |
| Hyperlipidemia | -0.023 | -0.060 (-0.145 to 0.025) | 0.164 | -0.066 | -0.200 (-0.232 to -0.167) | <0.001 |
| eGFR | -0.200 | -0.010 (-0.012 to -0.009) | <0.001 | -0.160 | -0.010 (-0.011 to -0.009) | <0.001 |
| Previous MI | 0.023 | 0.070 (-0.033 to 0.174) | 0.184 | -0.005 | -0.019 (-0.062 to 0.024) | 0.391 |
| Previous PCI/CABG | -0.028 | -0.098 (-0.214 to 0.019) | 0.099 | -0.049 | -0.186 (-0.230 to -0.141) | <0.001 |
| Heart failure | 0.050 | 0.189 (0.073 to 0.305) | 0.001 | 0.018 | 0.099 (0.044 to 0.154) | <0.001 |
| Atrial fibrillation | -0.008 | -0.023 (-0.108 to 0.062) | 0.595 | 0.034 | 0.161 (0.115 to 0.207) | <0.001 |
| Previous stroke | 0.033 | 0.129 (0.016 to 0.242) | 0.025 | 0.014 | 0.071 (0.022 to 0.120) | 0.005 |
| PAD | 0.056 | 0.237 (0.113 to 0.362) | <0.001 | 0.028 | 0.162 (0.106 to 0.218) | <0.001 |
| COPD | -0.025 | -0.084 (-0.182 to 0.014) | 0.094 | -0.011 | -0.059 (-0.109 to -0.008) | 0.023 |
| Previous/present cancer | -0.002 | -0.011 (-0.158 to 0.136) | 0.882 | -0.005 | -0.036 (-0.107 to 0.035) | 0.318 |
|  |  |  |  |  |  |  |

Analyses had been adjusted for all assessed variables, hospital and admission year (n=4367).

CI: confidence interval; eGFR: estimated glomerular filtration rate; MI: myocardial infarction; PCI: percutaneous coronary intervention; CABG: coronary artery bypass grafting; PAD: peripheral artery disease; COPD: chronic obstructive pulmonary disease.

**Supplemental Figure 1. Cumulative incidence of all-cause mortality in relation to hs-cTnT quartiles in patients with type 2 NSTEMI.**

**Supplemental Figure 2. Cumulative incidence of major adverse events in relation to hs-cTnT quartiles in patients with type 2 NSTEMI.**

MAE: major adverse events.
